# Medial opening low tibial osteotomy shifts the load laterally not only at the ankle joint but also at the knee joint

**DOI:** 10.1002/jeo2.70029

**Published:** 2024-12-17

**Authors:** Yoshihiro Wanezaki, Hiroaki Kurokawa, Yuki Ueno, Adrian Tablante, Nan Mei, Li Yinghao, Akira Taniguchi, Akemi Suzuki, Yuya Takakubo, Michiaki Takagi, Yasuhito Tanaka

**Affiliations:** ^1^ Department of Orthopedic Surgery Nara Medical University Kashihara city Nara Japan; ^2^ Department of Orthopedic Surgery Faculty of Medicine, Yamagata University Yamagata Japan

**Keywords:** ankle osteoarthritis, knee alignment, low tibial osteotomy, lower limb alignment

## Abstract

**Purpose:**

The purpose of this study was to determine the effects of medial opening low tibial osteotomy (LTO) on lower limb alignment, including the knee joint, 1 year after low tibial osteotomy.

**Methods:**

This study included 20 legs of 20 patients (mean age, 66.8 ± 5.4 years) who underwent LTO for medial ankle osteoarthritis and evaluated the changes in the hip–knee–ankle angle (HKA), percentage hip‐to‐ankle line (%HA), percentage hip‐to‐calcaneal line (%HC), medial proximal tibial angle (MPTA), knee joint line convergence angle (K‐JLCA), tibio‐calcaneal angle (TCA), tibial anterior surface angle (TAS), tibio‐plafond inclination (TPI), talar inclination (TI), ankle joint line convergence angle (A‐JLCA), mechanical ankle joint axis point (MAJAP) on radiographs and the Japanese Society for Surgery of the Foot (JSSF) ankle/hindfoot scale before and 1 year after low tibial osteotomy.

**Results:**

The mean preoperative/postoperative measured values showed the following: HKA (degrees) of 1.0 ± 3.7/−0.8 ± 3.7; %HC of 38.8 ± 10.0/53.8 ± 16.1; MPTA (degrees) of 85.6 ± 2.4/87.6 ± 2.1; and A‐JLCA (degrees) of 4.2 ± 2.9/1.1 ± 2.3 respectively. Including other measurements, a significant increase in the %HA, %HC, MPTA, TCA, TAS, MAJAP and JSSF ankle/hindfoot scale was observed postoperatively, whereas a significant decrease in the HKA, TPA, TI and A‐JLCA was observed postoperatively (*p* < 0.05). With the numbers available, no significant differences were observed between the preoperative and postoperative values of K‐JLCA (n.s.).

**Conclusion:**

After LTO, the entire lower limb alignment became valgus, and the loading points of the knee and ankle joints shifted laterally. These changes must be considered when performing LTO, especially in patients with lateral knee OA.

**Level of Evidence:**

Ⅳ

**Abbreviations**
A‐JLCA: ankle joint line convergence angle
HKA: hip–knee–ankle angle
HTO: high tibial osteotomy
ICC: intraclass correlation coefficients
JSSF: Japanese Society for Surgery of the Foot
K‐JLCA: knee joint line convergence angle
LTO: low tibial osteotomy
MAJAP: mechanical ankle joint axis point
MPTA: medial proximal tibial angle
OA: osteoarthritis
TAS: tibial anterior surface angle
TCA: tibio‐calcaneal angle
TI: talar inclination
TKA: total knee arthroplasty
TPI: tibio‐plafond inclination
TT: talar tilt
%HA: percentage hip‐to‐ankle line
%HC: percentage hip‐to‐calcaneal line

## INTRODUCTION

Approximately 6% of the population is affected by ankle osteoarthritis (OA) [6]. Ankle OA caused by trauma is more prevalent in Western countries. In contrast, primary ankle OA with no identifiable cause is more prevalent in Japan, which may be attributed to the tibial anterior surface angle (TAS) of patients in Japan being more varus than that in the patients in Western countries [25]. Evidence supporting the efficacy of conservative treatment for ankle OA, as well as the treatment options, remain limited [7, 29]. Surgical treatment strategies for ankle OA include osteotomy, ankle arthroplasty, and ankle arthrodesis. Osteotomy and ankle arthroplasty facilitate the preservation of joint motion; however, patient activity limits the indications for arthroplasty [20]. Medial opening low tibial osteotomy (LTO) is one of the osteotomies best indicated for malalignment corresponding to Takakura–Tanaka classification stage 3a with significant cartilage wear on the medial side only. As the number of active older adults increases in recent years with the ageing of society, the importance of osteotomy, similar to LTO, may increase owing to its ability to preserve joint motion [21, 24].

Knee OA, one of the most prevalent joint diseases in Japan, affects 35.2%, 48.2% and 51.6% of patients in their 60s, 70s and 80s in Japan, respectively [15, 30]. Previous studies have reported an association between knee OA and ankle OA [14, 18]. Total knee arthroplasty (TKA) and high tibial osteotomy (HTO) result in changes in ankle and hindfoot alignment in patients with knee OA postoperatively, impacting ankle and hindfoot symptoms [10, 24]. Therefore, a different preoperative plan, which considers the postoperative changes in the ankle and hindfoot alignment, must be formulated for patients undergoing HTO or TKA complicated ankle joint symptoms [1, 3].

It is important to understand the effects of LTO on the knee joints. However, no study has comprehensively examined the effect of LTO on lower limb alignment, including the knee joint. Therefore, this study aimed to determine the effect of LTO on lower limb alignment, including the knee joint. Our hypothesis is that LTO not only causes changes in the ankle joint alignment and loading point but also in the knee joint alignment and loading point.

## MATERIAL AND METHODS

**Figure 1:**
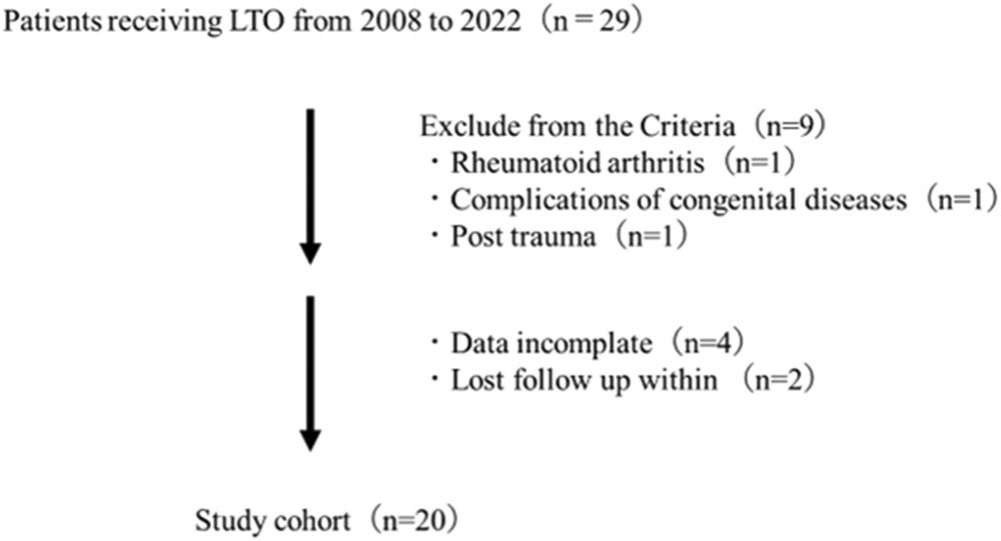
Flowchart of patients excluded from the analysis.

Patients who had undergone primary LTO for ankle OA at our hospital between 2008 and 2022 were included in this retrospective study. After excluding patients with neurological diseases, rheumatoid arthritis, congenital diseases, history of undergoing lower limb surgery, missing radiographs and other cases deemed unsuitable by the physician in charge, 20 legs of 20 patients (mean age at the time of surgery, 66.8 ± 5.4 years; mean body mass index, 24.4 ± 3.6 kg/m^2^) (Figure 1) were included. The study cohort comprised four male and 16 female participants. The Takakura–Tanaka classification [24] stages were 3a and 3b in 18 and two legs, respectively.

### Surgical technique

Weightbearing ankle radiographs were used for preoperative planning. Tibial osteotomy was performed 4–5 cm proximal to the inferior end of the medial malleolus. The target TAS was set as 96–98 degrees after correction via open wedge osteotomy. The surgery was performed with the patient in the supine position. The level of the tibial osteotomy was determined using the radiographs. The osteotomy level of the fibula was slightly proximal to that of the tibia. The fibula was osteotomized obliquely from the proximal posterior region to the distal anterior region. A Kirschner wire was inserted from the tip of the fibula and fixed similar to an intramedullary nail. Tibial osteotomy was performed vertically to the tibial axis, and the tibia was opened in accordance with the preoperative plan. A wedge‐shaped artificial bone was implanted into the osteotomy site and fixed with a locking plate. A below‐knee cast was used for 4–6 weeks postoperatively, followed by an ankle joint supporter for 8–12 weeks. Weightbearing was commenced gradually from the fourth postoperative week depending upon the pain, with the goal of achieving a full weightbearing gait at 6 weeks postoperatively. The Kirschner wire inserted into the fibula was removed 3–4 weeks postoperatively [21, 24].

### Radiographic technique and measurement parameters

Radiographic examinations were performed before and 1 year after LTO surgery. Whole‐leg and weightbearing subtalar radiographs were acquired for all patients. Weightbearing subtalar radiographs were acquired as described in previous reports [2, 5].

**Figure 2:**
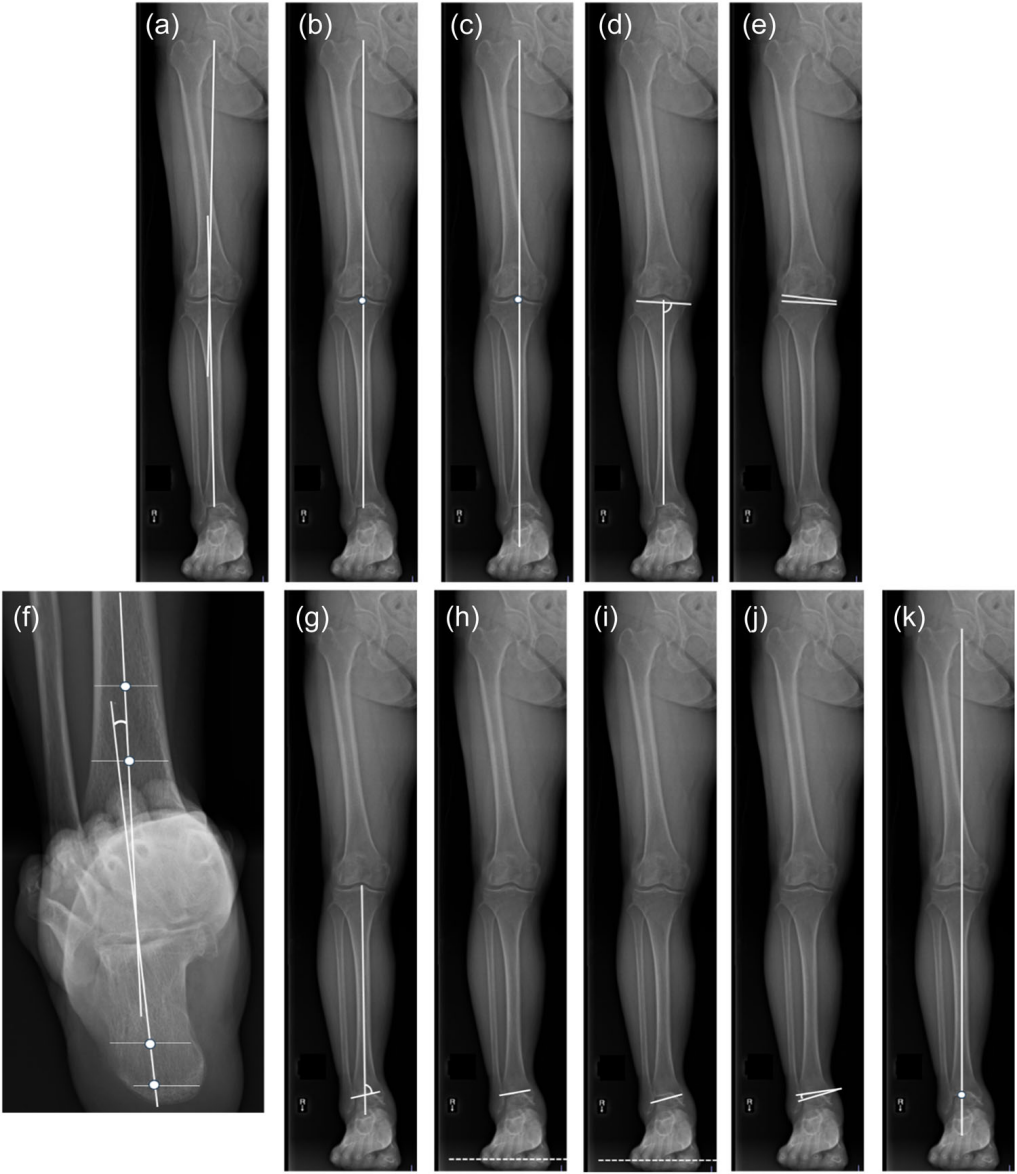
Measurement parameters. (a) Hip‐knee‐ankle angle (HKA); defined as the angle between the mechanical axes of the femur (centre of the femoral head to femoral intercondylar point) and tibia (tibial interspinous point to tibial mid‐plafond point), (b) % hip‐to‐ankle line (%HA); defined as the ratio of the distance between the point where the hip‐to‐ankle line passes and the medial edge of the tibia to the width of the tibial plateau, (c) % hip‐to‐calcaneus line (%HC); defined as the ratio of the distance between the point where the hip‐to‐calcaneal line passes and the medial edge of the tibia to the width of the tibial plateau, (d) medial proximal tibial angle (MPTA); defined as the angle between the mechanical axis and the proximal articular surface of the tibia, (e) knee joint line convergence angle (K‐JLCA); defined as the angle between the distal femoral and proximal tibial joint surfaces (positive for lateral opening), (f) tibiocalcaneal angle (TCA); defined as the angle between the tibial and calcaneal axes. The tibial axis was defined as the line between the midpoints of the tibial shaft 8 and 13 cm above the tip of the medial malleolus. The calcaneal axis was defined by two points. The first point was 7 mm proximal to the most distal part of the calcaneus on the horizontal line, which divided the bone at a 40:60 ratio, with the 40% segment beginning on the lateral side. The second point was at the middle of the horizontal line, 30 mm proximal to the most distal part of the calcaneus, (g) tibial anterior surface angle (TAS); defined as the angle between the lines drawn on the axis of the distal one‐third of the tibia and the plafond, (h) tibial plafond inclination (TPI); defined as the angle between the tibial plafond and the ground (positive for lateral inclination), (i) talar Inclination (TI); defined as the angle between the talar joint surface and the ground (positive for lateral inclination), (j) ankle joint line convergence angle (A‐JLCA); defined as the angle between the tibial plafond and the talar joint surface, (k) mechanical ankle joint axis point (MAJAP); defined as the percentage of the intersection of the tibial plafond with the line connecting the centre of the femoral head to the lowest point of the calcaneus to the entire tibial plafond.

The following parameters were measured: (1) Hip–knee–ankle angle (HKA), defined as the angle between the mechanical axes of the femur (centre of the femoral head to femoral intercondylar point) and tibia (tibial interspinous point to tibial mid‐plafond point) [14] (positive for varus). (2) Percentage hip‐to‐ankle line (%HA), defined as the ratio of the distance between the point where the hip‐to‐ankle line passes and the medial edge of the tibia to the width of the tibial plateau [23]. (3) Percentage hip‐to‐calcaneal line (%HC), defined as the ratio of the distance between the point where the hip‐to‐calcaneal line passes and the medial edge of the tibia to the width of the tibial plateau [8]. (4) Medial proximal tibial angle (MPTA), defined as the angle between the mechanical axis and the proximal articular surface of the tibia [19]. (5) Knee joint line convergence angle (K‐JLCA), defined as the angle between the distal femoral and proximal tibial joint surfaces (positive for lateral opening). (6) Tibio‐calcaneal angle (TCA), defined as the angle between the tibial and calcaneal axes. The tibial axis was defined as the line between the midpoints of the tibial shaft 8 and 13 cm above the tip of the medial malleolus. The calcaneal axis was defined by two points. The first point was 7 mm proximal to the most distal part of the calcaneus on the horizontal line, which divided the bone at a 40:60 ratio, with the 40% segment beginning on the lateral side. The second point was at the middle of the horizontal line, 30 mm proximal to the most distal part of the calcaneus [27] (positive for valgus). (7) TAS, defined as the angle between the lines drawn on the axis of the distal one‐third of the tibia and the plafond [25]. (8) Tibio‐plafond inclination (TPI), defined as the angle between the tibial plafond and the ground (positive for lateral inclination). (9) Talar inclination (TI), defined as the angle between the talar joint surface and the ground (positive for lateral inclination). (10) Ankle joint line convergence angle (A‐JLCA), defined as the angle between the tibial plafond and the talar joint surface [3, 22]. (11) Mechanical ankle joint axis point (MAJAP), defined as the percentage of the intersection of the tibial plafond with the line connecting the centre of the femoral head to the lowest point of the calcaneus to the entire tibial plafond [4] (Figure 2).

All image analyses were performed using SYNAPSE version 5 (FUJIFILM Medical System, Inc.). The Japanese Society for Surgery of the Foot (JSSF) ankle‐hindfoot scale was used for subjective evaluation preoperatively and 1 year postoperatively. This scale comprises three categories: pain (40 points), function (50 points) and alignment (10 points): total, 100 points [16, 17].

### Statistical analysis

All statistical analyses were performed using IBM SPSS Statistics for Windows version 25 (IBM). The normality of the data was evaluated using the Shapiro–Wilk test. Paired *t* test and Wilcoxon signed‐rank test were used to evaluate statistically significant differences. Statistical significance was set at *p* < 0.05. All radiographic assessments were performed twice by two researchers. The intra‐ and interobserver reliability were determined based on the intraclass correlation coefficients (ICC) of the radiographic assessments [21, 22]. ICCs were evaluated using ICC (1, 2) and ICC (2, 2) for intra‐ and interobserver reliabilities, respectively. The ICCs were classified as almost perfect (0.81–1.00), excellent (0.61–0.80), good (0.41–0.60) and slight (0.00–0.20), as described by Landis et al. [12]. The ICCs for intra‐ and interobserver reliabilities were greater than 0.76 (range, 0.76–0.99) for all measurements. The measurements obtained by one researcher were used in the analysis based on these results [12, 26, 27, 28]. The sample size was calculated using G*Power version 3.1.9. The significance level and power were set as 0.05 and 0.80, respectively, to analyze the difference between the two paired groups. Sixteen legs were included per group.

## RESULTS

**Figure 3:**
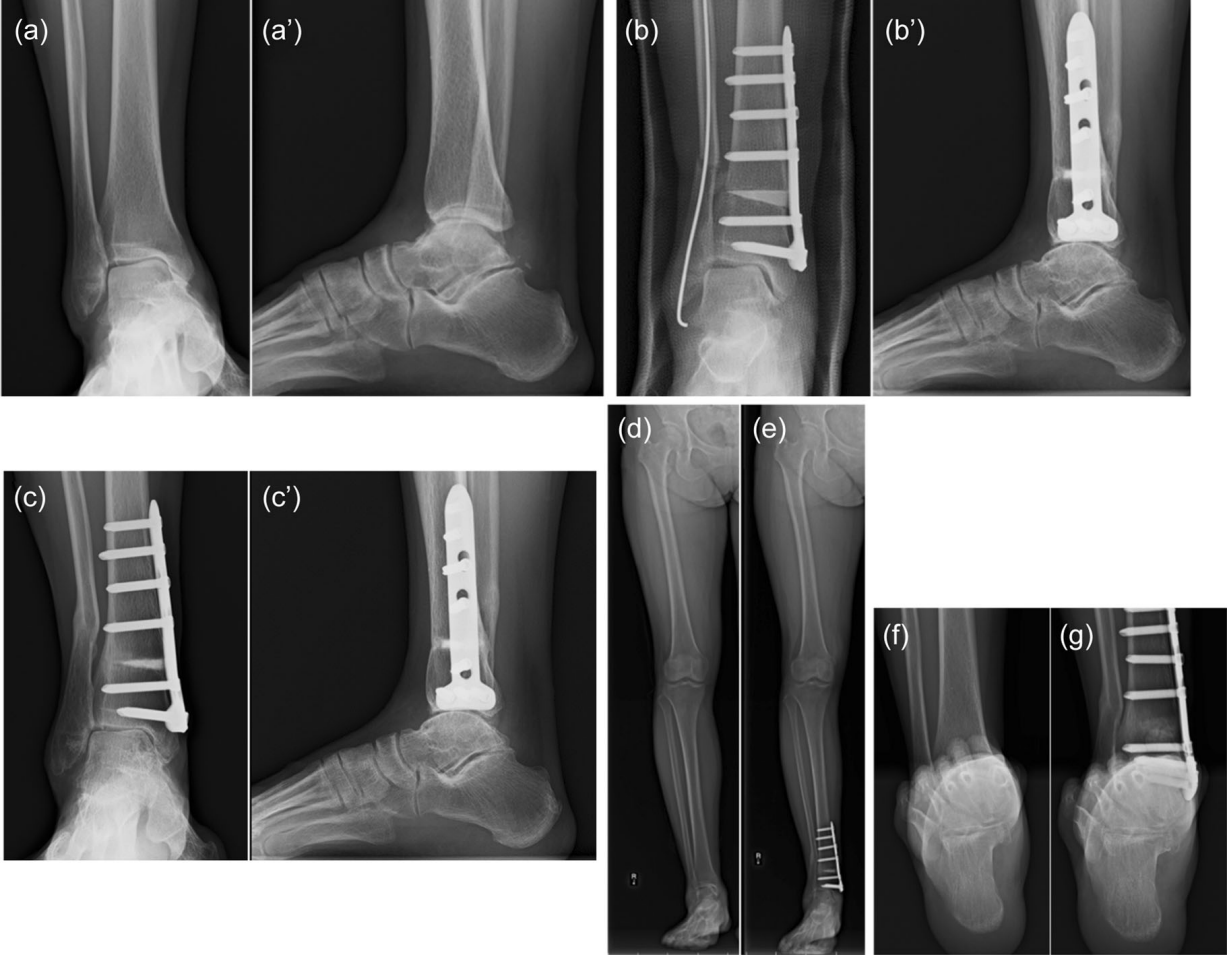
Case: 58‐year‐old female. (a and a') Preoperative radiograph of the frontal and lateral ankle joints. (b and b') Postoperative radiograph of the frontal and lateral ankle joints acquired immediately after the surgery. (c and c') Radiograph of the frontal and lateral ankle joints acquired 1 year postoperatively. (d) Radiograph of the whole leg standing radiography of preoperatively. (e) Radiograph of the whole leg standing radiography of 1 year postoperatively. (f) Weightbearing Subtalar radiograph acquired preoperatively. (g) Weightbearing Subtalar radiograph acquired 1 year postoperatively.

Radiographic measurement parameters are shown in Table 1. The mean change in each parameter is as follows: HKA was 1.8° of valgus, the knee loading point was 8.1% or 15% more lateralized, MPTA was 2.0° of varus, K‐JLCA was unchanged, TCA was 5.4° of valgus, TAS was 12.1° of valgus, TPI was 13.3° of valgus, TI was 16.4° of valgus, A‐JLCA decreased by 3.1° and MPJAP was 38.3° lateralized. The JSSF ankle/hindfoot scale pain, function, alignment and total scores were 15.3 ± 8.5/34.1 ± 6.0, 33.9 ± 7.0/46.8 ± 5.1, 5.9 ± 1.9/9.7 ± 1.2 and 55.1 ± 13.6/91.2 ± 8.1, respectively. In the statistical differences, %HA, %HC, MPTA, TCA, TAS, MAJAP and all the subscale and total JSSF ankle/hindfoot scale increased significantly postoperatively (*p* ＜ 0.05). With the numbers available, no significant differences were observed between the preoperative and postoperative values of K‐JLCA. The results showed that the lower limb alignment as a whole, including the hindfoot, became valgus and the loading points of the knee and ankle joints became more lateralized. The tilt of the ankle joint with relation to the ground was reduced. No obvious influence on the alignment of the knee joint surface was observed (Figure 3).

**Table 1:** Pre‐ and postoperative parameters.

	Preoperative	Postoperative	*p* Value
HKA (°)	1.0 ± 3.7	−0.8 ± 3.7	0.002
%HA	38.2 ± 8.3	47.5 ± 13.0	<0.001
%HC	38.8 ± 10.0	53.8 ± 16.1	<0.001
MPTA (°)	85.6 ± 2.4	87.6 ± 2.1	<0.001
K‐JLCA (°)	1.4 ± 1.1	1.4 ± 1.2	n.s.
TCA (°)	−2.5 ± 6.3	2.9 ± 4.0	<0.001
TAS (°)	85.5 ± 3.4	97.6 ± 2.5	<0.001
TPI (°)	6.3 ± 3.0	−7.0 ± 4.0	<0.001
TI (°)	10.5 ± 3.4	−5.9 ± 5.2	<0.001
A‐JLCA (°)	4.2 ± 2.9	1.1 ± 2.3	<0.001
MAJAP (%)	38.5 ± 15.9	76.8 ± 14.5	<0.001
JSSF ankle/hindfoot scale pain	15.3 ± 8.5	34.1 ± 6.0	<0.001
JSSF ankle/hindfoot scale function	33.9 ± 7.0	46.8 ± 5.1	<0.001
JSSF ankle/hindfoot scale alignment	5.9 ± 1.9	9.7 ± 1.2	<0.001
JSSF ankle/hindfoot scale total	55.1 ± 13.6	91.2 ± 8.1	<0.001

*Note*: Values are represented as mean ± SD.

Abbreviations: A‐JLCA, ankle joint line convergence angle; HKA, hip knee ankle angle; JSSF, Japanese Society for Surgery of the Foot; K‐JLCA, knee joint line convergence angle; MAJAP, mechanical ankle joint axis point; MPTA, medial proximal tibial angle; TAS, tibial anterior surface angle; TCA, tibiocalcaneal angle; TI, talar inclination; TPI, tibial plateau inclination; %HA, % hip‐to‐ankle line; %HC, % hip‐to‐calcaneus line.

## DISCUSSION

The most important findings of the present study were the identification of the effect of LTO on lower limb alignment, including the knee. After LTO surgery, the lower limb alignment as a whole becomes valgus and hindfoot alignment is closer to normal. The knee and ankle joint loading points are accordingly lateralized; however, this does not affect the knee surface alignment at the 1‐year follow‐up period. Several studies have investigated the effects of knee surgery on the foot. The ankle plane becomes parallel to the ground, and the hindfoot alignment improves to near‐normal values when TKA or HTO is performed to improve the varus knee alignment [3, 10, 13]. However, excessive correction of varus knee alignment can lead to overcorrection of ankle and hindfoot alignment and increased loading on the foot and hindfoot [3, 10, 13]. Thus, the symptoms associated with the foot must be considered preoperatively for knee surgery [3, 9, 10, 13]. However, the effect of LTO on lower limb alignment, including the knee joint, remains unclear.

The ideal TAS after LTO ranges from 96 to 98° [21, 24]. The mean postoperative TAS was 97.6° in the present study, indicating that good correction alignment was achieved. The JSSF ankle/hindfoot scale scores exhibited a significant improvement postoperatively. The MAJAP results revealed a shift in the load axis located on the medial side of the ankle joint to the lateral side. Moreover, TPI and TT exhibited a significant decrease postoperatively, whereas A‐JLCA exhibited a significant decrease postoperatively, indicating an improvement in ankle joint conformity.

LTO can be sufficiently corrective to the distal tibial articular surface, as the mean TAS became 12.1° valgus after LTO, but the effect on the tibial axis was approximately 2° of valgus, because mean HKA was 1.8° valgus and the mean MPTA was 2.0° valgus after LTO. In addition, %HA and %HC exhibited a significant increase, indicating that the knee joint load axis was lateralized postoperatively. With the numbers available, no significant differences were observed between %HA and %HC preoperatively in the present study; however, %HC was significantly greater than %HA postoperatively. %HC may be a more reliable load axis than %HA [8]. The differences between the postoperative %HA and %HC values observed in the present study may be attributed to the change of TCA to valgus after LTO. Changes in the hindfoot alignment reportedly cause differences in the %HC and %HA. Kim and colleagues reported that the hip to calcaneus line and hip to tatus line vary in patients with knee OA, which they considered to be attributed to the differences in the hindfoot alignment [11]. Choi and colleagues reported similar changes in the alignment of the hindfoot after LTO [19]. The change in TCA to valgus after LTO may have caused lateralization of the lowest point of the calcaneus. Thus, %HC was significantly higher than %HC postoperatively. The mean age before LTO surgery was 66.8 ± 5.4 years in the present study, and the prevalence of knee OA in this age group was over 35%; thus, the effect of LTO on the knee joint is very important [30]. The results of this study showed that both %HA and %HC were approximately 50%. Otsuka and colleagues recommended that the weightbearing axis be made neutral for highly active patients after HTO [18]. The change in the weightbearing axis in this study may have resulted in a change to a more natural loading axis for the knee joint as well. TKA for knee OA improved the symptoms of ankle OA owing to lateralization of the MAJAP in a previous study [26]. A similar effect can be achieved via LTO in patients with medial knee OA complications by lateralizing the load axis of the knee joint. The importance of changes in the load axis in the knee is evident from the concept of HTO. HTO, a surgery for medial‐type knee OA, has resulted in good outcomes due to its effect on reducing the load on the medial knee joint by lateralizing the axis of loading of the knee joint [22]. Furthermore, with the numbers available, no significant differences were observed in the K‐JLCA scales before and after LTO, indicating no progression of medial or lateral knee OA for at least 1 year postoperatively. Nevertheless, patients with lateral knee OA should be monitored as they are susceptible to experiencing worsening lateral knee joint pain after LTO. Furthermore, patients with progressive collapsing foot deformities whose TCA is highly valgus should be informed regarding the possibility of the TCA becoming more valgus after LTO.

The present study had certain limitations. First, the sample size of the present study was small. Second, the knee joint symptoms could not be evaluated, and detailed examinations other than radiography, such as the K‐JLCA, could not be performed. Thus, the assessment of the cartilaginous surface may not be accurate. Third, a two‐dimensional alignment evaluation was performed using radiographs in the present study; a three‐dimensional evaluation was not performed. Furthermore, the present study focused on evaluation of the whole lower limb in the coronal plane; evaluation in the sagittal plane was not considered. Fourth, the follow‐up period was only 1 year. However, this study is important in that it provides insights into the effects of LTO on the knee joints, which is crucial as society ages. Understanding that the knee joint loading point becomes more lateralized after LTO may help us confidently recommend surgery to patients planned for LTO, even if they have concomitant medial knee OA. Conversely, it is important to understand the possibility of increased pain on the lateral side of the knee joint when LTO is performed in patients with concomitant lateral knee OA.

## CONCLUSION

The present study demonstrated that after LTO, when the TAS was corrected to an average of 12.1° valgus, lower limb alignment became 1.8° valgus following HKA, the loading points of the knee and ankle joints were lateralized and the hindfoot alignment changed in the valgus direction. These changes must be considered when performing LTO in patients with lateral knee OA.

## AUTHOR CONTRIBUTIONS

All authors have (1) made substantial contributions to the study concept or the data analysis or interpretation; (2) drafted the manuscript or revised it critically; (3) approved the final version of the manuscript to be published; and (4) agreed to be accountable for all aspects of the work.

## CONFLICT OF INTEREST STATEMENT

The authors declare no conflict of interest.

## ETHICS STATEMENT

Ethical approval for this study was obtained from the Institutional Ethics Committee (approval number: IRB3724). All patients were informed that data from the case would be submitted for publication, and they provided their consent.

## Data Availability

N/A.
